# Spatio-Temporal Dynamics of African Swine Fever in Free-Ranging Wild Boar (*Sus scrofa*): Insights from Six Years of Surveillance and Control in Slovakia

**DOI:** 10.3390/vetsci12111027

**Published:** 2025-10-23

**Authors:** Peter Smolko, Jozef Bučko, Marek Štefanec, Tibor Lebocký, Martin Chudý, Rudolf Janto, Filip Kubek, Rudolf Kropil

**Affiliations:** 1 Department of Applied Zoology and Wildlife Management, Faculty of Forestry, Technical University in Zvolen, T. G. Masaryka 24, 960 01 Zvolen, Slovakia; xstefanec@is.tuzvo.sk (M.Š.); ylebocky@is.tuzvo.sk (T.L.); xkubek@is.tuzvo.sk (F.K.); kropil@is.tuzvo.sk (R.K.); 2 National Forest Centre, T.G. Masaryka 2175, 960 01 Zvolen, Slovakia; bucko@nlcsk.org; 3 Slovak Hunting Chamber, Štefánikova 10, 811 05 Bratislava, Slovakia; 4 State Veterinary and Food Administration of the Slovak Republic, Botanická 6190/17, 842 13 Bratislava, Slovakia; chudy@svpsr.sk (M.C.); janto@svpsr.sk (R.J.)

**Keywords:** surveillance, epidemiology, wild boar population, disease control, hunting management

## Abstract

**Simple Summary:**

This study analyzed six years of national monitoring data from Slovakia (2019–2024) to understand how ASF spread through wild boar populations and how it affected their numbers and management. The disease emerged during a period of record wild boar abundance, which allowed rapid expansion, particularly in eastern Slovakia, before moving westward. The combined effects of disease-related deaths and intensified hunting led to a substantial reduction in wild boar density throughout the country. Despite these declines, the virus continues to spread toward neighboring regions, emphasizing the need for coordinated control. The study highlights that maintaining low wild boar densities, promptly removing infected carcasses, and strengthening cooperation among hunters, veterinarians, and neighboring countries are essential to slow the spread of African swine fever. These insights support evidence-based management and contribute to the protection of both wildlife and domestic pig production in Europe.

**Abstract:**

African swine fever (ASF) has reshaped wild boar (*Sus scrofa*) populations and management across Europe since its reintroduction in 2007. ASF reached Slovakia in August 2019, when wild boar population size and harvest were at six-decade maximums. We analyzed data from six years (2019–2024) of national surveillance and control to quantify spatio-temporal ASF patterns in free-ranging wild boar. Using monthly virological (PCR) and serological (antibody) data from active (hunted) and passive (found dead) surveillance, we (1) estimated temporal variation in the effective reproduction number (R_t_); (2) modeled spatio-temporal prevalence in Slovakia and its eastern, central, and western regions; (3) linked these dynamics to management indicators such as wild boar density, harvest, and mortality; and (4) proposed measures to increase surveillance and control effectiveness. Passive surveillance showed greater diagnostic sensitivity than active surveillance for case detection (PCR: 46.5% vs. 0.48%; antibodies: 7.62% vs. 0.75%). R_t_ peaked at 3.83 in March 2021, then declined but periodically exceeded 1.0 through late 2024. Virological prevalence showed strong late-winter/early-spring seasonality and a persistent east-to-west gradient: peaks occurred first in the east (March 2021, March 2023), with the center surpassing the east in October 2023 and a subsequent rise in the west. Seroprevalence lagged and shifted westward later, peaking in March 2023 and increasing in western Slovakia from mid-2024. Wild boar density decreased by 36.3% from 2019 to 2024 and harvest-based density by 42.8%, returning to post-classical swine fever levels (2009–2013). We recommend prioritizing targeted carcass searches and rapid removal, maintaining low wild boar densities through sustained harvest of adult females, modernizing population monitoring methods, enhancing hunters’ compliance, and strengthening cross-border coordination to improve surveillance and control, thereby slowing ASF spread across Europe.

## 1. Introduction

African swine fever (ASF) is a highly contagious hemorrhagic viral disease affecting both wild and domestic suids. Domestic pigs (*Sus scrofa domesticus*) and Eurasian wild boar (*Sus scrofa*) are especially vulnerable, with infections leading to very high morbidity and mortality. Over the past two decades, ASF has become a major concern for Europe’s swine industry and wild boar management [1,2]. First identified in Kenya in 1921, ASF gradually spread to Europe via contaminated food products [3]. The first European incursion occurred in Portugal in 1957, followed by a re-emergence in 1960 that spread across the Iberian Peninsula and persisted until eradication in Portugal (1994) and Spain (1995), although ASF has remained endemic in Sardinia since 1978 [4]. After years of confinement mainly to Africa, the most significant reintroduction occurred in 2007 in Georgia, from where ASF spread through the Caucasus and the Russian Federation, marking the beginning of the current large-scale epidemic in Europe and beyond [1]. Since then, ASF has expanded rapidly across Eastern and Central Europe, reaching Poland by 2014 [5] and Hungary by 2018 [6]. The disease’s progression has caused severe socio-economic consequences, especially in countries heavily dependent on pork production, resulting in major losses due to culling and trade restrictions [1,7] (Gallardo et al., 2015; Sánchez-Cordón et al., 2018). The spread of ASF in wild boar has further complicated control efforts in free-ranging populations, forcing wildlife management and hunting practices across Europe to adopt enhanced surveillance, stricter biosecurity, and modified hunting regulations [8,9,10].

ASF likely entered Slovakia via infected wild boar from Hungary, as a positive case was detected in January 2019 approximately 400 m from the Slovak border. Subsequently, the first confirmed outbreak of ASF in Slovakia occurred in the southeastern part of the country in July 2019 in domestic pigs, about 500 m from the Hungarian border, followed by the first ASF-positive wild boar detected in August 2019, roughly 6 km from the infected pig farm [11]. In subsequent months, the virus spread progressively northward but mainly westward along the Hungarian border, and by late 2024 had reached western Slovakia [12]. This spread highlights the continuing challenge of ASF management in both domestic pigs and wild boar, underscoring the need for robust cross-border surveillance and biosecurity to prevent further transmission [13,14]. The course of the epidemic in Slovakia mirrors trends in other European countries, where ASF is primarily transmitted via wild boar movements with spillover to domestic pigs [15]. Continued surveillance and targeted management of wild boar populations remain essential for mitigating outbreaks and reducing impacts on wildlife management.

Wild boar populations in Slovakia have increased markedly over the past six decades, driven by favorable environmental conditions (e.g., intensive agriculture) and supplementary feeding in later decades, which have reduced winter mortality [16]. Population growth was slowed in the 1990s by control measures [17] and again during 2003–2008 by major outbreaks of classical swine fever (CSF), which temporarily reduced population growth and harvest numbers. However, when ASF emerged in 2019, wild boar numbers and harvest were at their maximum in six decades [18]. Since then, ASF has caused a substantial decline, reflecting both disease-driven mortality and intensified management aimed at limiting spread. These episodes illustrate the significant role of infectious diseases, especially ASF, in shaping wild boar dynamics and management strategies.

The primary objective of this study is to investigate the spatio-temporal dynamics of ASF in free-ranging wild boar in Slovakia, based on six years of surveillance and control data (2019–2024). Specifically, the goals of this study were to: (1) estimate the effective reproduction number (R_t_) and its temporal fluctuations, which provide insights into the progression and potential control of the disease within the wild boar population; (2) analyze spatio-temporal dynamics of ASF prevalence across Slovakia, focusing on its temporal and spatial variation between the main regions of the country (East, Center, and West); (3) assess the impact of ASF on Slovak wild boar population in terms of density, harvest and mortality; and (4) evaluate the effectiveness of surveillance and control measures implemented in Slovakia and provide recommendations for improving the management strategies to enhance ASF containment and prevention in the future. We believe this study contributes valuable insights into the epidemiology of ASF in wild boar populations, offering recommendations for improving surveillance, control measures, and management strategies to minimize the impact of ASF on wild boar populations throughout Europe.

## 2. Material and Methods

### 2.1. Study Area

**Figure 1:**
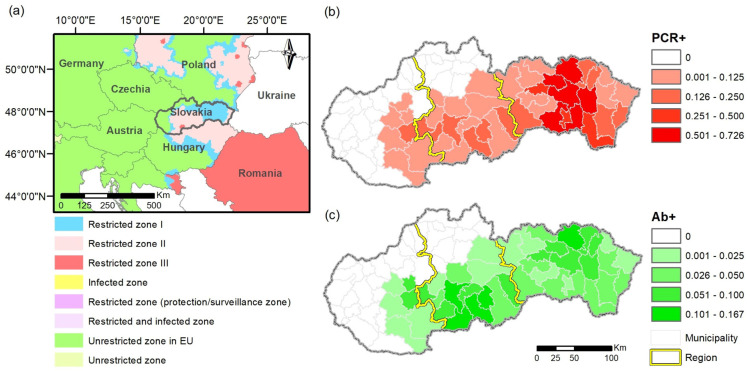
(**a**) Location of Slovakia within Europe and current EU ASF zoning, and (**b**) cumulative density (ind./km^2^) of virologically and (**c**) serologically positive ASF cases in Slovakia during 2019–2024.

The study was conducted in Slovakia (Figure 1), located in the temperate forests of the Western and partially Eastern Carpathians (48°47.687′–49°5.280′ N, 17°15.651′–22°33.929′ E; 94–2654 m a.s.l.). Slovakia covers a total area of 49,035 km^2^ and has a population of approximately 5.45 million, with an average density of 142 inhabitants per km^2^. The country has a temperate continental climate, characterized by warm summers and cold winters. Mean annual temperatures range from 5 to 10 °C, and average annual precipitation varies between 600 and 1200 mm, depending on altitude. Snow cover typically lasts from December to March, especially in higher elevations. Forests cover 41% of the territory, with deciduous forests dominating at lower elevations (<800 m a.s.l.), accounting for 26% of the land area. These are mainly composed of oak (*Quercus* spp.) and beech (*Fagus sylvatica*), with admixtures of hornbeam (*Carpinus betulus*), maple (*Acer* spp.), and ash (*Fraxinus excelsior*). Forests in higher elevations (800–1000 m a.s.l.) are primarily coniferous (15%), dominated by Norway spruce (*Picea abies*), with admixtures of silver fir (*Abies alba*), European larch (*Larix decidua*), and Scots pine (*Pinus sylvestris*). The remaining landscape consists of shrubland (4.5%), open meadows (6%), agricultural land (43%) including pastures and crop fields (mainly wheat, oats, and corn), and urbanized areas (5.5%) [19].

Wild boar is widely distributed across Slovakia, inhabiting all regions from lowlands to mountainous areas up to 1200 m.a.s.l., but it is most abundant in warmer southern forest-agriculture mosaic landscapes, which provide abundant foraging resources and cover. Other wild ungulates present in the Slovakia include red deer (*Cervus elaphus*), roe deer (*Capreolus capreolus*), fallow deer (*Dama dama*), and mouflon (*Ovis musimon*) [20]. Slovakia’s mountainous regions also support stable populations of large carnivores, such as brown bear (*Ursus arctos*), Eurasian lynx (*Lynx lynx*) and grey wolf (*Canis lupus*), which selects for wild boar as their main prey [21].

### 2.2. Wild Boar Management in Slovakia

**Figure 2:**
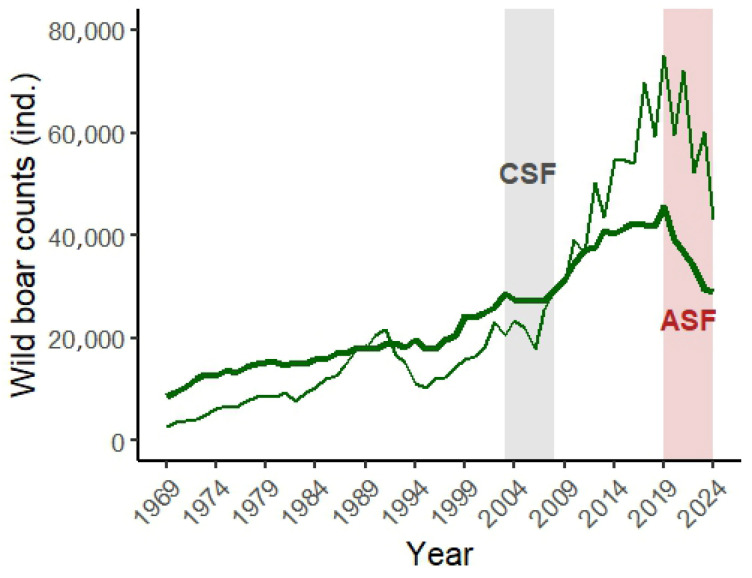
Long-term trend in wild boar pre-harvest counts (thick line) and harvest (thin line) in Slovakia from 1969 to 2024, highlighting the effects of classical swine fever (CSF; 2003–2008) and African swine fever (ASF; 2019–present) on the wild boar population and its management.

Wild boar numbers have increased significantly over recent decades in Slovakia, facilitated by milder winters, supplementary feeding, and changes in land use that favored population growth. Population control has been primarily implemented through regulated hunting, with harvest levels closely following spring count estimates. Management strategies have been influenced by disease outbreaks, notably CSF between 2003 and 2008 and ASF since 2019. These epizootics triggered targeted interventions, including intensified culling, biosecurity measures, and movement restrictions. Long-term trends in wild boar counts and harvests are monitored annually by the Slovak Hunting Chamber and the National Forest Centre and form the basis for assessing the impacts of management and disease on population dynamics (Figure 2).

Wild boar can be legally hunted in Slovakia year-round, with specific seasonal and demographic restrictions outlined in national hunting legislation (Act No. 274/2009 Coll. on Hunting). Adult wild boars (male and female) are typically hunted from July to end of February, while juveniles and piglets may be hunted throughout the year. Legal hunting methods include individual hunting (stalking, stand hunting) and driven hunts, which are typically organized in the autumn and winter months. Additionally, night hunting with artificial light or thermal optics is permitted under certain conditions, particularly in regions affected by ASF, to reduce population densities and mitigate disease spread. All harvested animals must be reported and examined for ASF as part of the national surveillance program (Figure 1).

### 2.3. ASF Surveillance Data

Monthly data from each municipality on the number of samples tested for ASF in Slovakia from 2019 to 2024, including both virological (PCR) and serological, resp. antibody (Ab) tests, were collected through active (hunting) and passive (found dead) surveillance as part of the National Eradication Plan for ASF in Slovakia 2020–2025 (Table 1). Data on annual spring counts, hunting data, and wild boar mortality records per municipality (Figure 1) were obtained from the Integrated System of Hunting and Population Management [18].

### 2.4. Estimation of the Effective Reproduction Number (R_t_)

To assess the temporal dynamics of ASF transmission in wild boar, we estimated the time-varying effective reproduction number (R_t_) within a Bayesian framework using the EpiEstim package [22] in R 4.1.3 [23]. The analysis was based on monthly ASF incidence data (laboratory-confirmed PCR-positive wild boar) collected in Slovakia between 2019 and 2024, with each month treated as a discrete time step in the incidence series. R_t_ at time *t* was estimated as the ratio of observed incidence (Iₜ) to the total infectiousness (Λ_*t*_), where Λₜ represents the contribution of past cases weighted by the serial interval (SI) distribution. Specifically,

$$\Lambda_{t}=\sum_{s=1}^{t-1}I_{t-s}w_{s}$$

with *w*_*s*_; denoting the probability that a secondary case occurs *s* time units after a primary case, derived from the SI distribution. Incidence was assumed to follow a Poisson process, and the posterior distribution of R_t_ was obtained from a Gamma prior, updated over a sliding window of length τ. We applied a parametric gamma-distributed SI with a mean of 1.5 months and a standard deviation of 0.5 months, reflecting ASF incubation and virus persistence in wild boar carcasses [8,24]. Estimation was initiated once the cumulative incidence exceeded 40 cases (January 2020). For interpretation, values of R_t_ > 1 indicate sustained epidemic growth, while R_t_ < 1 suggests declining transmission. Parts of the R coding and language editing were supported by the AI-based language model ChatGPT 5.0 [25].

### 2.5. Spatio-Temporal Dynamics in ASF Prevalence

We modeled the temporal trend in ASF virological and serological prevalence using monthly positive and negative data from wild boar harvested or found dead from 2019 to 2024 in Slovakia. Prevalence values were ln-transformed and Generalized Additive Model (GAM) was used to model the epidemic progression over time. First, we used year and month of surveillance as explanatory variables to describe overall ASF dynamics in Slovakia. Next, we split data into Eastern, Central and Western regions of Slovakia and used it as a categorical variable in our next set of models to evaluate the temporal ASF dynamics within each region. For this purpose, we used an interaction term of the date and region in GAMs. We use the term prevalence; however, we acknowledge that our data document apparent prevalence [26], which is positively correlated with disease incidence [27].

### 2.6. Impact of the ASF on Wild Boar Management

The impact of ASF on wild boar management in Slovakia was assessed using annual hunting reports on spring population counts, harvest, and mortality. We compared wild boar management data from the ASF-affected period (2019–2024) with the preceding 10-year baseline (2009–2018). First, we calculated wild boar density per 1 km^2^ of hunting area from the pre-harvest counts and from harvest counts, i.e., post-CSF period (2009–2013) and pre-ASF period (2014–2018) and compared mean density with following years (2019–2024) using Tukey’s HSD test. The pre-harvest count was calculated as spring counts multiplied by the growth rate of 1.5 (Act No. 274/2009 Coll. on Hunting). Next, we assessed hunting intensity by calculating the harvest: pre-harvest count ratio, stratified by age and sex categories (adult males, adult females, subadults, and piglets), and compared these ratios between periods without ASF (2009–2018) and after ASF introduction (2019–2024) using t-test. Finally, we estimated mortality rates as the proportion of wild boar found dead relative to pre-harvest population for each sex/age category to evaluate changes before (2009–2018) and after ASF introduction (2019–2024) using Tukey’s HSD test (*p* ≤ 0.05).

## 3. Results

**Figure 3:**
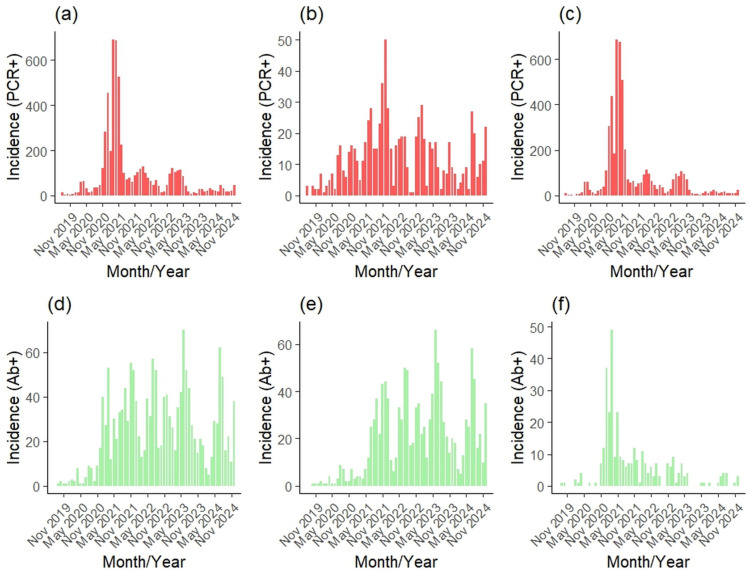
Monthly numbers of positively tested wild boar for ASF virus (red) and antibodies (green) during overall (**a**,**d**), active (**b**,**e**), and passive (**c**,**f**) monitoring in Slovakia during 2019–2024.

We analyzed virological data from 177,236 wild boars either harvested (active surveillance) or found dead (passive surveillance) in Slovakia between 2019 and 2024 (Table 1). Of these, 167,147 were sampled through active surveillance and 10,940 through passive surveillance. A total of 5855 individuals tested positive for ASF (Figure 3a), with 809 from active surveillance (Figure 3b) and 5085 from passive surveillance (Figure 3c), resulting in an overall PCR-based ASF prevalence of 3.30% (active: 0.48%; passive: 46.48%). We also analyzed serological data from 170,583 wild boars collected during the same period (active: 167,076; passive: 4330). Among these, 1584 tested positive for ASF antibodies (Figure 3d), with 1256 cases from active (Figure 3e) and 330 from passive surveillance (Figure 3f), corresponding to an overall seroprevalence of 0.93% (active: 0.75%; passive: 7.62%).

### 3.1. Effective Reproduction Number (R_t_)

**Figure 4:**
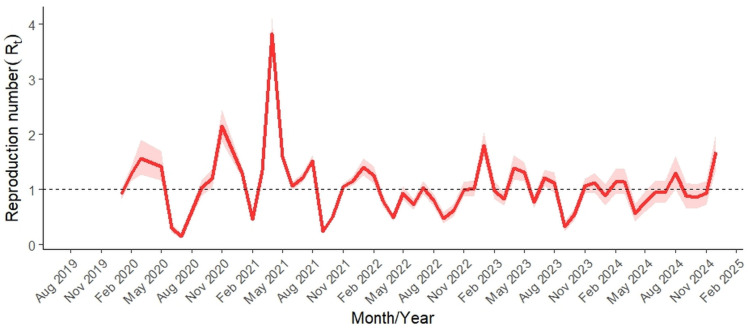
Estimated time-varying effective reproduction number (R_t_; mean ± 95% Credible interval) during the ASF epidemic in wild boar population in Slovakia, 2019–2024. Estimation was initiated after ≥40 positive cases had been reached (January 2020).

The estimated time-varying reproduction number (R_t_) of ASF in wild boar fluctuated markedly between August 2019 and December 2024 (Figure 4). During the early epidemic wave, R_t_ exceeded 1.0, reaching 2.15 (mean; 95% CrI: 1.87–2.45) in November 2020, and peaking at 3.83 (95% CrI: 3.56–4.10) in April 2021. Although the magnitude generally declined after 2021, several short-term increases persisted, including 1.80 (95% CrI: 1.59–2.03) in January 2023 and 1.67 (95% CrI: 1.36–2.01) in December 2024. Uncertainty bands (95% CrI) were wider during the early epidemic phase and in months with low incidence, indicating reduced precision in these periods.

### 3.2. Spatio-Temporal Dynamics in ASF Prevalence in Slovakia

**Figure 5:**
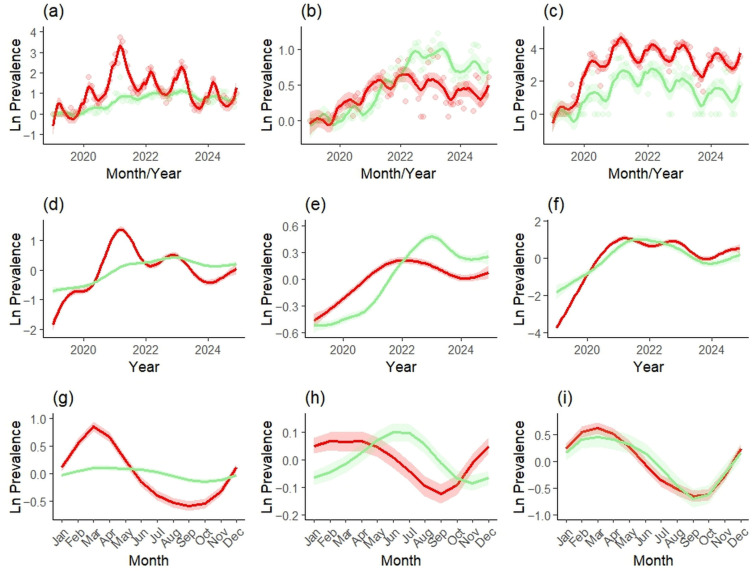
Predicted viro-(red) and sero-(green) prevalence (Ln-transformed) by the overall (**a**,**d**,**g**), active (**b**,**e**,**h**) and passive monitoring (**c**,**f**,**i**) in Slovakia during 2019–2024. Top row shows predictions compared to real data (**a**–**c**), middle row shows the annual effect (**d**–**f**) and bottom row shows the seasonal—annually repeating effect (**g**–**i**).

The GAM revealed clear temporal trends in both virological and serological ASF prevalence in Slovakia during 2019–2024 (Table S1 and Table 2; Figure 5). Virological prevalence showed a pronounced peak in March 2021 (Figure 5d), reaching 28.0% (95% CI: 21.3–36.8%), followed by a decline in 2022 and a subsequent rise to 10.2% (7.8–13.4%) in March 2023 (Figure 5a). Serological prevalence increased more gradually, peaking at 3.1% (2.6–3.6%) in March 2023, then slowly declined through 2024.

Patterns differed between surveillance strategies. In active monitoring, virological prevalence peaked at 1.9% (1.7–2.1%) in February 2022 and declined thereafter (Figure 5b). In passive monitoring, it reached 94.8% (74.4–100%) in March 2021 before dropping sharply (Figure 5c). Serological prevalence in active monitoring peaked at 2.8% (2.5–3.1%) in June 2023, while in passive monitoring it peaked at 15.6% (9.1–26.9%) in March 2022.

Seasonal effects were strongest in passive virological surveillance (Figure 5i), with prevalence highest in late winter–spring (March), lowest in late summer–early autumn (September) and increasing again in late autumn (Figure 5g). This seasonal cycle was weaker in active monitoring (Figure 5h) and almost absent in serological data (Figure 5g).

### 3.3. Regional Differences in ASF Prevalence

**Figure 6:**
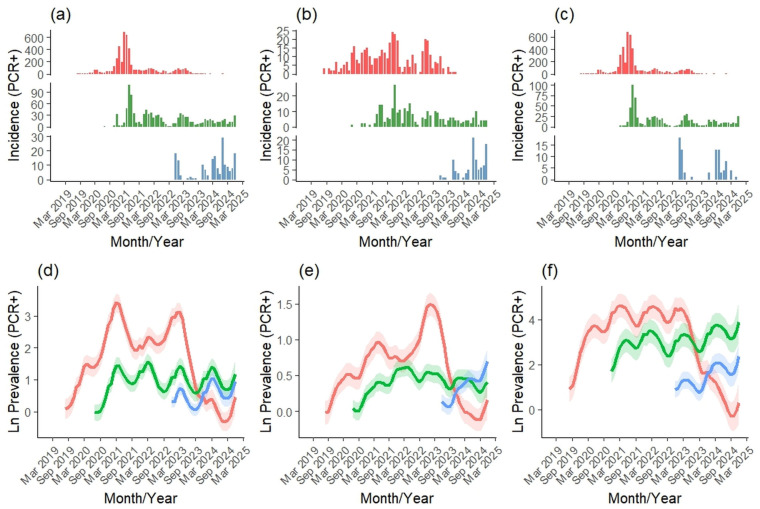
Virological (PCR+) ASF incidence and prevalence within eastern (red), central (green) and western (blue) Slovakia during 2019–2024 by the overall (**a**,**d**), active (**b**,**e**) and passive (**c**,**f**) monitoring.

**Figure 7:**
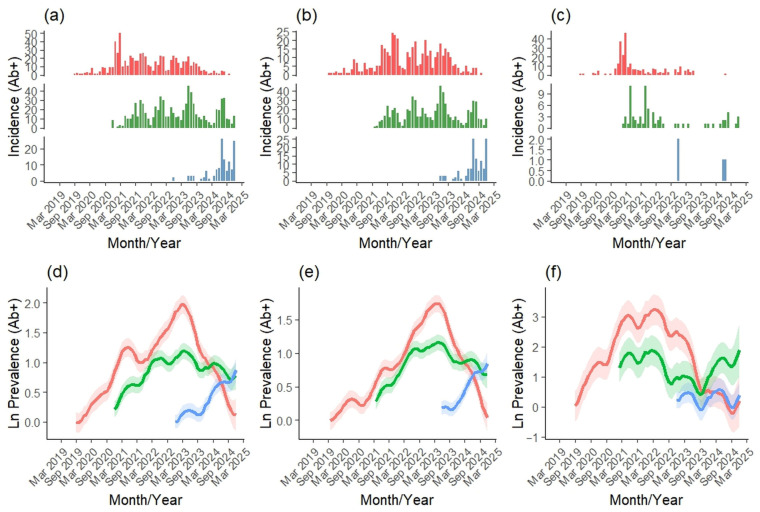
Serological (Ab+) ASF incidence and prevalence within eastern (red), central (green) and western (blue) Slovakia during 2019–2024 by the overall (**a**,**d**), active (**b**,**e**) and passive (**d**,**f**) monitoring.

ASF virological and serological prevalence showed clear regional differences across Slovakia during 2019–2024 (Figure 6 and Figure 7). Prevalence was highest in eastern Slovakia, with major peaks in March 2021 and March 2023, exceeding levels in central and western regions until October 2023, when central Slovakia surpassed the east, followed by a rise in the west in November 2023. Active and passive monitoring revealed similar trends, though active data indicated that western prevalence exceeded central levels by the end of 2024. These patterns align with the established east–west spread of ASF, originating in eastern Slovakia, progressing through the central region, and reaching the west by late 2022, with the east–west gradient most pronounced in passive surveillance.

Serological prevalence showed a clear east–west gradient, with eastern Slovakia maintaining the highest levels until early 2024 (Figure 7d–f). It peaked in March 2023, followed by a sharp decline, while central Slovakia’s prevalence continued to rise, surpassing the east in March 2024. Western Slovakia remained low for most of the period but increased rapidly from mid-2024, overtaking the central region in August 2024. Active and passive monitoring revealed the same spatial pattern, though differences between regions were most pronounced in passive surveillance.

### 3.4. Impact of ASF on Wild Boar Management in Slovakia

**Figure 8:**
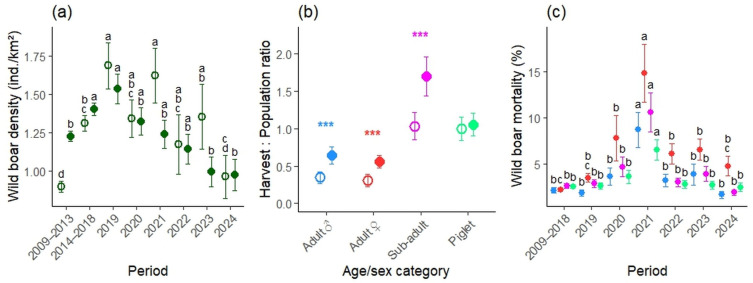
(**a**) Average wild boar density (mean ± SE) in Slovakia calculated from pre-hunting population estimates (solid circles) and from realized harvest (open circles) for the post-CSF period (2009–2013) and the pre-ASF period (2014–2018), as well as for individual years during ASF presence (2019–2024). Different letters indicate significant differences in density between periods (Tukey HSD test, *p* ≤ 0.05); periods sharing the same letter are not significantly different. (**b**) Harvest intensity, expressed as the ratio of harvest to the pre-harvest population (mean ± SE), during the disease-free decade (2009–2018; open circles) and in years with ASF presence (2019–2024; solid circles) within each social category. Asterisks denote significant differences between periods (*t*-test, *** *p* ≤ 0.001). (**c**) Average mortality (mean ± SE) of adult males (blue), adult females (red), subadults (purple), and piglets (green) in Slovakia during the disease-free period (2009–2018) and in years with ASF presence (2019–2024). Different letters indicate significant differences in mortality between periods within each social category (Tukey HSD test, *p* ≤ 0.05); periods within a social category sharing the same letter are not significantly different.

Wild boar density in Slovakia varied significantly across periods (Figure 8a). Densities were lowest in the post-CSF period (2009–2013), increased during the pre-ASF period (2014–2018), particularly in harvest-based estimates, and peaked historically in 2019 before steadily declining by 36.3% resp. by 42.8% to post-CSF levels by 2024. During 2019–2024, harvest numbers exceeded pre-harvest population estimates, raising concerns about the accuracy of recent survey methods. Harvest intensity (Figure 8b) was significantly higher in ASF years than in the disease-free decade across all social categories, with the largest increase in adult males by 86.42%, adult females by 81.33%, sub-adults by 64.09% and piglets by 5.49%. Mortality (Figure 8c) increased significantly in 2021 in all social groups, especially among adult females, then declined but remained above pre-ASF levels for all categories.

## 4. Discussion

This study provides the first comprehensive assessment of the spatio-temporal dynamics of ASF and its impacts on free-ranging wild boar population and management in Slovakia. ASF emerged in 2019 during a period of historically highest wild boar abundance (Figure 2), which facilitated rapid spread (March 2021) and led to high early prevalence (April 2021), particularly in eastern Slovakia. We observed pronounced temporal patterns (both annual and seasonal) in virological and serological prevalence at the national level (Figure 5), as well as significant regional differences (Figure 6 and Figure 7). Passive surveillance proved far more effective than active surveillance, detecting virologically positive animals at 97× the rate (46.48% vs. 0.48%) and seropositive animals at more than 10× the rate (7.62% vs. 0.75%). The markedly higher ASF detection rate in passive surveillance reflects its greater diagnostic sensitivity, since carcasses mostly represent animals that died from the disease, whereas hunted individuals come from the apparently healthy population segment. ASF caused a significant increase in mortality, which, together with an >80% increase in adult harvest, resulted in a 36% reduction in pre-harvest wild boar density and a 43% reduction in harvest density in Slovakia (Figure 8). These declines were followed by a subsequent drop in ASF incidence, especially in eastern Slovakia, likely due to reduced contact rates and therefore limited transmission in the remaining population [28]. Nevertheless, ASF continues to advance westward toward Austria and Czechia, highlighting the need for coordinated cross-border management.

Although estimation of the effective reproduction number is fundamental for understanding the status of an epidemic, studies specifically addressing this epidemiological parameter remain relatively scarce [29,30]. At the country level, R_t_ showed considerable temporal variability, peaking at 3.83 (95% CrI: 3.56–4.10) in March 2021, but generally declining thereafter apart from seasonal fluctuations driven by seasonal peaks in wild boar contact rates, delays in carcass removal, or westward spread into naïve subpopulations [31]. Similar R_t_ values and the overall trends were observed also in Dominican Republic [30] and Sardinia, Italy [29]. Although the overall pattern suggests that the current ecological dynamics of ASF in Slovakia resemble an endemic state, continued vigilance is needed, as R_t_ in late 2024 suggest potential for renewed epidemic growth in western Slovakia and further progression towards Czech and Austrian borders [32].

Spatio-temporal analysis confirmed a strong east–west gradient in ASF prevalence. The highest virological prevalence occurred in eastern Slovakia in March 2021 and March 2023, with passive surveillance detecting particularly high values consistent with severe disease outcomes [2]. Serological prevalence increased more slowly, reflecting delayed antibody development in surviving animals [29], and later shifted westward, peaking in central and eventually western Slovakia in 2024. Seasonality strongly influenced ASF prevalence, especially in passive surveillance, underscoring the role of environmental persistence and wild boar behavior in transmission [28,33]. Winter peaks may reflect both increased contact rates during the mating season and slower carcass decomposition, which prolongs virus persistence [34]. In contrast, higher summer temperatures accelerate decomposition and reduce ASF stability [35,36], limiting transmission rate. Consistently, a recent analysis across multiple EU countries showed ASF cases in wild boar commonly peak in winter and early spring (December–March), indicating increased transmission during this period [31].

ASF had a profound effect on wild boar management in Slovakia. Population density in 2024 decreased by 36.3% to 0.98 ind./km^2^ compared to 2019, and harvest-based density decreased by 42.8% to 0.96 ind./km^2^, with 2024 levels of harvest matching those of the post-CSF period (2009–2013). Similar and even higher reductions in wild boar populations were observed throughout Europe. For example, in Estonia, wild boar density declined from ~0.56 wild boar/km^2^ in 2012/13–2015/16 to 0.10 wild boar/km^2^ in 2016/17, and further to 0.07 wild boar/km^2^ in 2017/18, representing an overall reduction of nearly 90% following ASF emergence [37]. A similar trend was reported in Lithuania, where densities decreased from ~27 to ~7 wild boar per km^2^, corresponding to a 60–75% reduction attributed to ASF impacts and associated management interventions [38]. Harvest intensity increased markedly during ASF years, particularly in adults (>80%), consistent with management strategies aimed at reducing recruitment [39]. Mortality peaked in 2021 and remained above pre-ASF levels. Because Europe-wide assessments report no consistent sex- or age-specific mortality [2,40], the apparent female-biased peak in our dataset likely reflects reporting bias, i.e., including deliberate misclassification, such as recording subadults as adult females to avoid culling sows, rather than biology. Hunters are often reluctant to shoot adult sows because they drive future recruitment [41]; however, sparing adult females can accelerate population rebound and elevate the risk of renewed ASF waves in recovering populations [42].

### Management Implications

Based on our results and the current ASF situation in Europe, we propose the following recommendations to increase the effectiveness of control measures and slow the spread of ASF across the continent:

1. Maintaining low wild boar densities: ASF-related mortality combined with control measures in Slovakia has reduced wild boar densities to 0.98 ind./km^2^ and harvest density to 0.96 ind./km^2^, levels last recorded more than a decade ago. According to the European Food Safety Authority [32], maintaining wild boar densities below ~1 ind./km^2^ substantially reduces contact rates between individuals and thereby limits ASF transmission. Sustained hunting pressure, particularly on adult females, will therefore be essential to prevent rapid population recovery and to mitigate future outbreaks.
2. Maintaining effective ASF surveillance: Surveillance should remain intensive in western Slovakia, where ASF prevalence has recently increased and where high wild boar densities combined with pig farming could facilitate rapid amplification. Early detection in this region requires continued targeted carcass searches, robust passive surveillance, and rapid carcass disposal to minimize environmental virus persistence. In the future, adaptive sampling strategies based on surveillance weights could be implemented to optimize efficiency and reduce overall monitoring costs.
3. Increasing hunters’ compliance with control measures: Enhancing compliance is critical for ASF management. Financial incentives for hunters, together with stricter enforcement of regulations, may improve adherence to control measures and increase their effectiveness.
4. Improving surveillance methods for wild boar population estimates: Traditional survey-based spring counts likely underestimate true population size, as harvest often exceeded pre-harvest estimates. Modernized census methods such as camera trapping, genetic mark–recapture, or drone-based surveys should be integrated to provide more accurate estimates.
5. Improving scientific knowledge on wild boar population and movement dynamics: Improved data on wild boar social structure, reproduction, survival, and movement (e.g., daily ranges, corridors, and barriers) are scarce and needed to inform local targeted control and resource allocation. Such knowledge would enhance the effectiveness of ASF management strategies.
6. Enhancing cross-border coordination of stakeholders: Effective ASF control requires close collaboration among stakeholders and authorities across national borders. At the EU level, this coordination is supported by harmonized frameworks such as Regulation (EU) 2023/594, which establishes common rules for ASF surveillance, zoning, and movement restrictions for pigs, wild boar, and related products to limit transboundary spread. These measures are complemented by the Standing Group of Experts on ASF in Europe under the GF-TADs initiative, which facilitates the exchange of epidemiological information and best practices among affected countries. Combining such coordinated frameworks with sustained population control, enhanced passive surveillance, and improved population estimation methods will strengthen Europe’s capacity to contain ASF and protect both wildlife and domestic pig production.

## 5. Conclusions

This study provides the first comprehensive assessment of African swine fever (ASF) dynamics and impacts on wild boar populations in Slovakia. ASF emerged during peak wild boar abundance, enabling rapid spread and high early prevalence. Following ASF-related mortality and intensified harvest reduced wild boar densities by over one-third. Despite subsequent declines in incidence, ASF continues to expand westward, posing risks to neighboring countries. Maintaining densities below 1 individual/km^2^, sustaining intensive passive surveillance, and improving hunter compliance are essential to limit transmission and prevent resurgence. Modernized population monitoring and stronger cross-border coordination under EU frameworks will further enhance Europe’s capacity to contain ASF and safeguard both wildlife and domestic pig production.

## Figures and Tables

**Table 1:** Number of ASF-negative and -positive wild boar tested for a presence of virus (PCR) and antibodies (Ab) in Slovakia during 2019–2024, and the ASF prevalence (%) within the overall, active and passive monitoring.

Test/ Year		Overall ASF Monitoring			Active ASF Monitoring			Passive ASF Monitoring		
		ASF−	ASF+	Prev.	ASF−	ASF+	Prev.	ASF−	ASF+	Prev.
	2019	34,925	32	0.1	33,514	10	0.0	1415	22	1.5
	2020	29,657	738	2.4	29,024	98	0.3	1066	679	38.9
	2021	33,225	3283	9.0	32,105	250	0.8	1493	3033	67.0
PCR	2022	23,212	817	3.4	22,520	173	0.8	692	644	48.2
	2023	26,917	679	2.5	26,209	151	0.6	707	528	42.8
	2024	23,445	306	1.3	22,966	127	0.5	482	179	27.1
	∑	**171,381**	**5855**		**166,338**	**809**		**5855**	**5085**	
	2019	34,331	5	0.0	33,515	3	0.0	820	2	0.2
	2020	29,348	66	0.2	29,071	40	0.1	728	28	3.7
	2021	32,900	430	1.3	32,105	232	0.7	1159	198	14.6
Ab	2022	22,801	384	1.7	22,349	329	1.5	452	55	10.8
	2023	26,449	400	1.5	25,969	370	1.4	479	30	5.9
	2024	23,170	299	1.3	22,811	282	1.2	362	17	4.5
	∑	**168,999**	**1584**		**165,820**	**1256**		**4000**	**330**	

**Table 2:** Parameter estimates of the top generalized additive models describing African swine fever (ASF) prevalence in the wild boar population in Slovakia during 2019–2024, based on (a) overall, (b) active, and (c) passive monitoring. β—regression coefficient; edf > 1 indicates a non-linear trend (when a smoothing terms s() or te() were applied); t and F represent the test statistics for the t- and F-tests, respectively.

Test	Model	Smooth Term	Overall Monitoring		Active Monitoring		Passive Monitoring	
			β/edf	t/F	β/edf	t/F	β/edf	t/F
PCR	Slovakia	s(Date)	8.4	42.64 ***	4.4	17.09 ***	7.22	68.60 ***
		s(Month)	5.13	16.07 ***	2.83	1.37 **	4.18	7.81 ***
		R^2^	83.4		58.9		79.4	
	Regional	Region: East	1.43	29.74 ***	0.54	20.78 ***	2.75	27.87 ***
		Region: Center	0.72	−10.49 ***	0.30	−6.51.51 ***	2.13	−4.48 ***
		Region: West	0.19	−18.27 ***	0.09	−12.23 ***	0.57	−15.64 ***
		te(Date, Region) East	8.34	59.65 ***	8.33	34.59 ***	6.42	37.44 ***
		te(Date, Region) Center	5.83	14.82 ***	4.49	12.34 ***	6.17	29.52 ***
		te(Date, Region) West	2.46	11.52 ***	3.45	11.70 ***	2.56	17.83 ***
		s(Month)	4.21	9.86 ***	3.42	1.92 ***	3.35	3.22 ***
		R^2^	90.1		73.1		89.6	
Ab	Slovakia	s(Date)	5.89	28.17 ***	6.01	52.48 ***	4.83	11.32 ***
		s(Month)	2.53	1.24 **	2.61	1.48 ***	2.97	1.86 ***
		R^2^	77.3		84.6		53.3	
	Regional	Region: East	0.86	31.58 ***	0.71	31.60 ***	1.49	14.70 ***
		Region: Center	0.59	10.49 ***	0.53	−5.42 ***	0.90	−4.08 ***
		Region: West	0.12	18.27 ***	0.13	−18.51 ***	0.12	−9.60 ***
		te(Date, Region) East	7.82	56.40 ***	7.65	73.72 ***	5.33	20.05 ***
		te(Date, Region) Center	5.12	41.67 ***	6.20	54.79 ***	5.35	7.31 ***
		te(Date, Region) West	4.02	16.36 ***	4.40	21.78 ***	1.00	2.16
		s(Month)	3.02	2.23 ***	2.83	1.91 ***	2.82	1.61 ***
		R^2^	84.9		87.7		56.6	

Note: “***” *p* ≤ 0.001; “**” *p* ≤ 0.01.

## Data Availability

The datasets presented in this article are not readily available because they are the property of the State Veterinary and Food Administration of the Slovak Republic and the National Forest Centre. Requests to access the datasets should be directed to the State Veterinary and Food Administration of the Slovak Republic and the National Forest Centre.

## Supplementary Materials

The following supporting information can be downloaded at: https://www.mdpi.com/article/10.3390/vetsci12111027/s1, Table S1: Candidate models for ASF virological (PCR) and serological (Ab) prevalence from the overall, active (hunting) and passive (found dead) monitoring of wild boar population in Slovakia during 2019–2024.

## References

[1] Sánchez-Cordón P.J., Montoya M., Reis A.L., Dixon L.K. (2018). African swine fever: A re-emerging viral disease threatening the global pig industry. Vet. J..

[2] Sauter-Louis C., Conraths F.J., Probst C., Blohm U., Schulz K., Sehl J., Fischer M., Forth J.H., Zani L., Depner K. (2021). African Swine Fever in Wild Boar in Europe—A Review. Viruses.

[3] Penrith M.L., Kivaria F.M. (2022). One hundred years of African swine fever in Africa: Where have we been, where are we now, where are we going?. Transbound. Emerg. Dis..

[4] Luisa D.M., Luisa M.M., Simona I., Paolo T., Paolo C., Francesco F. (2020). African Swine Fever: Lessons to Learn From Past Eradication Experiences. A Systematic Review. Front. Vet. Sci..

[5] Frant M.P., Gal-Cisoń A., Bocian Ł., Ziętek-Barszcz A., Niemczuk K., Szczotka-Bochniarz A. (2022). African Swine Fever (ASF) Trend Analysis in Wild Boar in Poland (2014–2020). Animals.

[6] Náhlik A., Erdélyi K., Bálint A., Tari T. African Swine Fever (ASF) in Hungary—History of the First Year After the Outbreak. Proceedings of the 34th International Union of Game Biologists (IUGB) Congress.

[7] Gallardo M.C., de la Torre Reoyo A., Fernández-Pinero J., Iglesias I., Muñoz M.J., Arias M.L. (2015). African swine fever: A global view of the current challenge. Porc. Health Manag..

[8] Guberti V., Khomenko S., Masiulis M., Kerba S., Pittiglio C. (2019). African Swine Fever in Wild Boar: Ecology and Biosecurity. FAO Animal Production and Health Manual.

[9] Ferran J., Giovanna M., Licoppe A., Francisco R.-F., Annick L., Petr V., Erika C., Carme R. (2021). Management of wild boar populations in the European Union before and during the ASF crisis. Understanding and Combatting African Swine Fever. An European Perspective.

[10] Migliore S., Hussein H.A., Galluzzo P., Puleio R., Loria G.R. (2023). African Swine Fever and Its Control Measures in Wild Boar: A “De Iure Condito” Analysis in the European Union. Animals.

[11] State Veterinary and Food Administration of the Slovak Republic (2020). Národný Eradikačný Program pre Africký mor Ošípaných v Diviačej Populácii na Slovensku v Roku 2020 [National Eradication Plan for African Swine Fever in Wild Boar Population in Slovakia in 2020].

[12] State Veterinary and Food Administration of the Slovak Republic (2025). Národný Eradikačný Program pre Africký mor Ošípaných v Diviačej Populácii na Slovensku v Roku 2025 [National Eradication Plan for African Swine Fever in Wild Boar Population in Slovakia in 2025].

[13] Beltran-Alcrudo D., Falco J.R., Raizman E., Dietze K. (2019). Transboundary spread of pig diseases: The role of international trade and travel. BMC Vet. Res..

[14] Oelke J., Jarynowski A. (2025). Sovereignty Beyond the Human: ASF in the German-Polish Borderland. DIE ERDE—J. Geogr. Soc. Berl..

[15] Nielsen S.S., Alvarez J., Bicout D.J., Calistri P., Depner K., Drewe J.A., Garin-Bastuji B., Gonzales Rojas J.L., EFSA (European Food Safety Authority), EFSA Panel on Animal Health and Welfare (AHAW) (2021). Scientific Opinion on the assessment of the control measures of the category A diseases of Animal Health Law: African Swine Fever. EFSA J..

[16] Massei G., Kindberg J., Licoppe A., Gačić D., Šprem N., Kamler J., Baubet E., Hohmann U., Monaco A., Ozoliņš J. (2015). Wild boar populations up, numbers of hunters down? A review of trends and implications for Europe. Pest Manag. Sci..

[17] Štofík J., Bučko J. (2020). Zmeny v poľovnom manažmente najvýznamnejších druhov poľovnej zveri na slovensku po II. Svetovej vojne (1949–2015)/Changes of the hunting management of the most important ungulate species in Slovakia after World War II. (1949–2015). Acta Fac. For..

[18] NFC SR (National Forest Centre of the Slovak Republic) (2025). Integrated System of Hunting and Population Management (ISLHP) Database.

[19] CORINE Land Cover 2018 European Copernicus Programme. https://land.copernicus.eu/en/products/corine-land-cover.

[20] Smolko P., Garaj P., Lebocký T., Bútora Ľ., Pataky T., Jaňáková Z., Babic M., Veselovská A., Kubala J., Kropil R. (2022). Soil nutrients and deer density affect antler size of the Carpathian red deer. Mamm. Biol..

[21] Guimarães N.F., Álvares F., Ďurová J., Urban P., Bučko J., Iľko T., Brndiar J., Štofik J., Pataky T., Barančeková M. (2022). What drives wolf preference towards wild ungulates? Insights from a multi-prey system in the Slovak Carpathians. PLoS ONE.

[22] Cori A., Ferguson N.M., Fraser C., Cauchemez S. (2013). A new framework and software to estimate time-varying reproduction numbers during epidemics. Am. J. Epidemiol..

[23] R Core Team (2022). R (Version 4.1.3): A Language and Environment for Statistical Computing. R Foundation for Statistical Computing.

[24] Lange M., Guberti V., Thulke H.H. (2017). Understanding ASF spread and persistence in wild boar populations: A modeling approach. Prev. Vet. Med..

[25] OpenAI (2025). ChatGPT.

[26] Greiner M., Gardner I.A. (2000). Epidemiologic issues in the validation of veterinary diagnostic tests. Prev. Vet. Med..

[27] Miller M.W., Wolfe L.L. (2021). Inferring Chronic Wasting Disease Incidence from Prevalence Data. J. Wildl. Dis..

[28] Nielsen S.S., Alvarez J., Bicout D.J., Calistri P., Depner K., Drewe J.A., Garin-Bastuji B., Gonzales Rojas J.L., Gortazar Schmidt C., EFSA (European Food Safety Authority) (2021). ASF Exit Strategy: Providing cumulative evidence of the absence of African swine fever virus circulation in wild boar populations using standard surveillance measures. EFSA J..

[29] Loi F., Cappai S., Laddomada A., Feliziani F., Oggiano A., Franzoni G., Rolesu S., Guberti V. (2020). Mathematical Approach to Estimating the Main Epidemiological Parameters of African Swine Fever in Wild Boar. Vaccines.

[30] Schambow R.A., Carrasquillo N., Kreindel S., Perez A.M. (2025). An update on active and passive surveillance for African swine fever in the Dominican Republic. Sci. Rep..

[31] Rogoll L., Güttner A.K., Schulz K., Bergmann H., Staubach C., Conraths F.J., Sauter-Louis C. (2023). Seasonal Occurrence of African Swine Fever in Wild Boar and Domestic Pigs in EU Member States. Viruses.

[32] Ståhl K., Boklund A.E., Podgórski T., Vergne T., Aminalragia-Giamini R., Abrahantes J.C., Papaleo S., Mur L., EFSA (European Food Safety Authority) (2025). Epidemiological analysis of African swine fever in the European Union during 2024. EFSA J..

[33] Bergmann H., Schulz K., Conraths F.J., Sauter-Louis C. (2021). A Review of Environmental Risk Factors for African Swine Fever in European Wild Boar. Animals.

[34] Pepin K.M., Golnar A.J., Abdo Z., Podgórski T. (2020). Ecological drivers of African swine fever virus persistence in wild boar populations: Insight for control. Ecol. Evol..

[35] Olesen A.S., Lohse L., Boklund A., Halasa T., Belsham G.J., Rasmussen T.B., Bøtner A. (2018). Short time window for transmissibility of African swine fever virus from a contaminated environment. Transbound. Emerg. Dis..

[36] Mazur-Panasiuk N., Wozniakowski G. (2020). Natural inactivation of African swine fever virus in tissues: Influence of temperature and environmental conditions on virus survival. Vet. Microbiol..

[37] Schulz K., Oļševskis E., Viltrop A., Masiulis M., Staubach C., Nurmoja I., Lamberga K., Seržants M., Malakauskas A., Conraths F.J. (2022). Eight Years of African Swine Fever in the Baltic States: Epidemiological Reflections. Pathogens.

[38] Tarvydas A., Belova O. (2022). Effect of Wild Boar (*Sus scrofa* L.) on Forests, Agricultural Lands and Population Management in Lithuania. Diversity.

[39] Mačiulskis P., Masiulis M., Pridotkas G., Buitkuvienė J., Jurgelevičius V., Jacevičienė I., Zagrabskaitė R., Zani L., Pilevičienė S. (2020). The African Swine Fever Epidemic in Wild Boar (*Sus scrofa*) in Lithuania (2014–2018). Vet. Sci..

[40] Chenais E., Depner K., Guberti V., Dietze K., Viltrop A., Ståhl K. (2019). Epidemiological considerations on African swine fever in Europe 2014–2018. Porc. Health Manag..

[41] Bieber C., Ruf T. (2005). Population dynamics in wild boar Sus scrofa: Ecology, elasticity of growth rate and implications for the management of pulsed resource consumers. J. Appl. Ecol..

[42] Bartoš L., Turek K., Křístek Š., Bartošova J. (2025). Wild Boar Paradox—Intensive Hunting Boosts Population Increase. JOJ Wildl. Biodivers..

