# Supplementary material for: Spatio-Temporal Dynamics of African Swine Fever in Free-Ranging Wild Boar (Sus scrofa): Insights from Six Years of Surveillance and Control in Slovakia
Source: Vet Sci. 2025 Oct 23;12(11):1027. doi: 10.3390/vetsci12111027 (PMC12656868; doi:10.3390/vetsci12111027)
Supplement: Supplementary file 1 [file vetsci-12-01027-s001.zip › vetsci-3894154-supplementary.pdf]

SUPPLEMENTARY MATERIAL

Table. S1. Candidate models for ASF virological (PCR) and serological (Ab) prevalence from the overall, active (hunting) and passive (found dead) monitoring of wild boar population in Slovakia during 2019–2024.

Test	Model	Structure	Overall monitoring				Active monitoring				Passive monitoring			
			k	LL	ΔAIC	w	k	LL	ΔAIC	w	k	LL	ΔAIC	w
PCR	Slovakia	Date + Month	16.2	-10.0	**0.0**	**1.0**	11.1	29.0	**0.0**	**1.0**	14.6	-34.4	**0.0**	**1.0**
		Date	9.4	-62.0	90.3	0.0	6.8	20.2	8.8	0.0	8.5	-67.9	54.6	0.0
		Null	2.0	-93.2	138.0	0.0	2.0	-6.9	53.5	0.0	2.0	-122.1	150.2	0.0
	Regional	Date + Month + Region + Date*Region	27.3	-100.5	**0.0**	**1.0**	26.7	30.9	**0.0**	**1.0**	25.0	-256.9	**0.0**	**1.0**
		Date + Month + Region	6.0	-264.4	285.3	0.0	6.0	-90.2	200.8	0.0	6.0	-390.2	228.4	0.0
		Date + Month	4.0	-299.5	351.4	0.0	3.0	-114.7	243.8	0.0	4.0	-425.6	295.3	0.0
		Date	3.0	-303.0	356.5	0.0	4.0	-114.2	244.7	0.0	3.0	-427.3	296.7	0.0
		Null	2.0	-306.5	361.4	0.0	2.0	-122.4	257.1	0.0	2.0	-438.6	317.2	0.0
Ab	Slovakia	Date + Month	12.4	10.8	**0.0**	**1.0**	12.1	36.1	**0.0**	**1.0**	11.5	-83.8	**0.0**	**1.0**
		Date	7.9	1.1	10.5	0.0	8.1	26.4	11.4	0.0	7.0	-95.3	14.1	0.0
		Null	2.0	-42.6	86.0	0.0	2.0	-35.9	123.9	0.0	2.0	-115.4	44.2	0.0
	Regional	Date + Month + Region + Date*Region	25.9	21.2	**0.0**	**1.0**	27.3	65.8	**0.0**	**1.0**	20.7	-264.6	**0.0**	**1.0**
		Date + Month + Region	6.0	-119.0	240.4	0.0	6.0	-93.4	275.6	0.0	6.0	-337.8	117.0	0.0
		Date + Month	4.0	-164.7	327.9	0.0	3.0	-132.9	348.7	0.0	3.0	-361.1	157.7	0.0
		Date	3.0	-166.6	329.7	0.0	4.0	-132.2	349.2	0.0	4.0	-360.8	159.0	0.0
		Null	2.0	-194.8	384.0	0.0	2.0	-172.5	425.9	0.0	2.0	-363.4	160.2	0.0
